# Cooper on the Testis

**Published:** 1845-03

**Authors:** 


### COOPER ON THE TESTIS.

The two great works of Sir Astley Cooper, "The Structure and Diseases of the Testis," and the "Thymus Gland," have been issued by Lea & Blanchard in a volume of large size and great beauty. As a specimen of typography, it is one of the most creditable that has yet come from the American press. As a work of science, it is esteemed one of the most able of the many able productions of its eminent author. No authority in surgery is higher than that of Sir Astley Cooper, and in this elegant volume it will be found that he embraced, ten years ago, nearly all that is known in regard to the anatomy and surgery of the testis. It is to be hoped that the enterprising publishers will be encouraged to continue the publication of the works of Sir Astley in this style. Y.

